# Assessing the effects of prenatal poly-drug exposure on fetal brain vasculature using optical coherence angiography

**DOI:** 10.1117/1.JBO.28.7.076002

**Published:** 2023-07-18

**Authors:** Raksha Raghunathan, Jessica Gutierrez, Chih-Hao Liu, Manmohan Singh, Rajesh C. Miranda, Kirill V. Larin

**Affiliations:** a University of Houston, Department of Biomedical Engineering, Houston, Texas, United States; b Weill Cornell Medicine, Houston Methodist Cancer Center, Department of Systems Medicine and Bioengineering, Houston, Texas, United States; c TAMHSC College of Medicine, Department of Neuroscience and Experimental Therapeutics, Bryan, Texas, United States

**Keywords:** brain vasculature, ethanol, nicotine, varenicline, murine fetus, optical coherence angiography

## Abstract

**Significance:**

Maternal exposure to drugs during pregnancy is known to have detrimental effects on the fetus. Alcohol (ethanol) and nicotine are two of the most commonly co-abused substances during pregnancy, and prenatal poly-drug exposure is common due, in part, to the prevalence of unplanned pregnancies. The second trimester is a critical period for fetal neurogenesis and angiogenesis. When drug exposure occurs during this time, fetal brain development is affected. Several behavioral, morphological, and functional studies have evaluated the changes in fetal brain development due to exposure to these drugs individually. However, research on the combined effects of ethanol and nicotine is far more limited, specifically on fetal vasculature changes and development.

**Aim:**

We use correlation mapping optical coherence angiography (cm-OCA) to evaluate acute changes in fetal brain vasculature caused by maternal exposure to a combination of ethanol and nicotine.

**Approach:**

Ethanol (16.6% v/v, at a dose of 0.75g/kg) and nicotine (at a dose of 0.1  mg/kg) were administered to pregnant mice after initial cm-OCA measurements *in utero*. Subsequent measurements were taken at 5-min intervals for a total period of 45 min. Results from these experiments were compared to results from our previous studies in which the mother was exposed to only ethanol (dose: 0.75  g/kg) or nicotine (dose: 0.1  mg/kg).

**Results:**

While results from exposure to ethanol or nicotine independently showed vasoconstriction, no significant change in vasculature was observed with combined exposure.

**Conclusion:**

Results suggested antagonistic effects of ethanol and nicotine on fetal brain vasculature.

## 1 Introduction

Poly-drug use refers to the use of two or more drugs together or one after the other within a short period of time. In 2019, almost half of overdose deaths involved poly-drug use.^1^ The short-term and long-term effects of poly-drug use depend on various factors, including the drugs used, their type and combination, the doses consumed, and the health (including size and weight) of the individual consuming them. Ethanol and nicotine are two of the most commonly co-abused substances. Around 20% of the adult population in the United States have reported simultaneous use of ethanol and nicotine.^2^^,^^3^ This co-dependency could be due to psychosocial, pharmacological, or molecular factors.^4^ Although the sites of action of ethanol and nicotine are different, the interactions between the effects of ethanol and nicotine are still being investigated.^4^ While some acute effects of these drugs, such as relaxation, reward, and analgesia, are similar and could be synergistic when the drugs are used concurrently, some other effects of nicotine may antagonize certain effects of acute ethanol exposure.^4^

Due to the relatively high percentage of women who have reported ethanol or nicotine use during pregnancy, the concern about the co-abuse of these drugs is high in pregnant women. Moreover, nearly half of the reported pregnancies were unplanned in the United States in 2011.^5^ Due to the prevalence of unplanned pregnancies, poly-drug abuse can easily continue as the pregnancy progresses to the second trimester, which is the critical period for fetal neurogenesis and angiogenesis.^6^ The vasculature that develops during this period is known to support various critical processes of development.^7^^–^^9^ Hence, it is necessary to study the effects of prenatal poly-drug use during the second trimester of pregnancy. While studies have been performed to understand the effects of prenatal exposure to the co-abuse of ethanol and nicotine at the molecular and behavioral levels,^10^^–^^13^ less research has focused on vasculature changes in the fetal brain.

Histological staining, ultrasound biomicroscopy, micro-computed tomography, and micro-magnetic resonance imaging have been used for small animal embryonic imaging.^14^ However, these methods are limited for live embryonic imaging due to limitations in imaging depth, low resolution, invasiveness, imaging speed, reliance on external contrast agents, and the need for ionizing radiation. Recently, photoacoustic imaging has been utilized to assess the ethanol-induced vasculature changes in the fetal brain, but this work did not include the influence of poly-drug exposure.^15^ Optical coherence tomography (OCT)^16^ has been successfully used for live imaging of small animal embryonic development over the past decade.^17^^–^^19^ Its noninvasive nature, ability to provide live cross-sectional images with no external contrast agents, and relatively high temporal and spatial resolutions have quickly made OCT a sought-after imaging technique for live embryonic imaging.^20^^–^^24^ We have used OCT to study various aspects of mouse and rat embryonic development *in utero*, thus demonstrating its capability of live embryonic imaging.^25^^–^^30^ While OCT was introduced as a structural imaging modality, the development of functional extensions of OCT has broadened its applications. One such functional extension is angiographic OCT, which was developed to image microvasculature and blood flow.^31^^–^^36^

This study used correlation mapping optical coherence angiography (cm-OCA),^37^ a type of angiographic OCT, to evaluate acute changes in fetal brain vasculature due to prenatal exposure to a combination of ethanol and nicotine, during the first-to-second trimester equivalent period in a mouse model *in utero*. Results showed that in comparison to the groups that were exposed to ethanol and nicotine independently, the group that was exposed to a combination of ethanol and nicotine did not show any significant change over time.

## 2 Materials and Methods

### 2.1 OCT System

OCT images of the fetal brain were acquired using a phase-stabilized swept source OCT system. The system consisted of a broadband swept source laser with a central wavelength of 1310 nm, a scan range of 150 nm, and a scan rate of 30 kHz. The system had a transverse resolution of 16  μm, axial resolution of 11  μm in air, incident power on the sample of 11 mW, and sensitivity of ∼98.5  dB. The interference pattern was recorded by a balanced photodetector, and a high-speed analog-to-digital converter was used for digitizing the spectral interference pattern. More information on this system can be found in our previous work.^38^^–^^42^

### 2.2 Animal Manipulations and Dosing

The animal manipulation procedure was similar to our previous work.^39^^–^^42^ Overnight mating was set up with CD-1 mice, and the presence of a vaginal plug was considered gestational day (GD) 0.5. On GD 14.5, the pregnant mice were anesthetized through isoflurane inhalation and placed on a heated surgical platform to maintain body temperature. Abdominal hair was removed, and a small incision was made in the abdomen, exposing the uterine horn for imaging. The embryo selected for imaging was stabilized using forceps, and initial OCT measurements were taken. The mother was administered the respective drugs for the study via intragastric gavage, and subsequent OCT measurements were taken for a total period of 45 min at 5-min intervals. The uterus was hydrated with 1X phosphate-buffered saline 1 min before every measurement. The mouse was euthanized at the end of the experiment through isoflurane overdose, followed by cervical dislocation. All procedures were performed under an approved protocol by the University of Houston Institutional Animal Care and Use Committee.

For the first study, pregnant mice (N=5) were administered a combination of ethanol and nicotine at doses of 0.75  g/kg and 0.1  mg/kg, respectively. Doses that caused minimal to moderate effects were selected based on our previous dose-response studies.^41^^,^^42^ Results from these experiments were compared to results from our previous work, where ethanol and nicotine were administered independently.^41^^,^^42^

### 2.3 Studying the Combined Effect of Ethanol and Varenicline

Varenicline is a partial nicotinic acetylcholine receptor agonist that is used for smoking cessation in humans.^43^ Varenicline produces a moderate level of receptor stimulation and lower sustained levels of dopamine release. This reduces the symptoms of nicotine withdrawal and helps with nicotine cessation. Since varenicline produces a moderate level of nicotinic receptor stimulation, it was crucial to test the combined effects of varenicline (as a replacement to nicotine) with ethanol, as the effects of varenicline for smoking cessation in pregnant women are not yet completely known.

For this study, pregnant mice (N=7) were administered a combination of ethanol and varenicline at doses of 0.75  g/kg and 0.1  mg/kg, respectively, to match the ethanol and nicotine experimental group. Results from this group were compared to the group that was administered a combination of ethanol and nicotine and the groups that were administered ethanol and nicotine individually.

### 2.4 Imaging, Quantifications, and Statistics

Each 3D OCT dataset acquired consisted of 600 B-scans, and each B-scan consisted of 600 A-scans. Five B-scans were recorded at each spatial position to obtain the angiographic OCT data.^37^ The total area scanned was ∼6.0  mm×6.2  mm, and the total acquisition time for each dataset was 84 ms, including the scanning mirror flyback time. The cm-OCA algorithm^37^^,^^44^^,^^45^ used to obtain the 3D vasculature maps and the remaining data processing steps were similar to our previous work.^40^^–^^42^

Maximum intensity projections (MIPs) of 3D cm-OCA images were calculated to obtain *en face* images. These images were used to perform quantifications. Amira software (EFI Co., Portland, Oregon, United States) was used for final denoising and to perform the vessel diameter (VD) quantifications. All the quantifications were performed on the main branch of the vessel. The results shown in this study include results from our previously published work.^41^^,^^42^

First, four nonparametric Kruskal–Wallis analyses of variance (ANOVAs) were performed to assess the changes in vasculature over time for the four different groups (ethanol, nicotine, ethanol+nicotine,ethanol+varenicline). Next, a two-sided Mann–Whitney U test was performed to assess the statistical significance between each of the groups with the independent drug exposures and combined drug exposures at 45 min post-exposure. Thus, there were a total of six Mann–Whitney tests that were performed. Bonferroni correction was performed to correct for multiple testing for the pair-wise tests.

## 3 Results

**Fig. 1:**
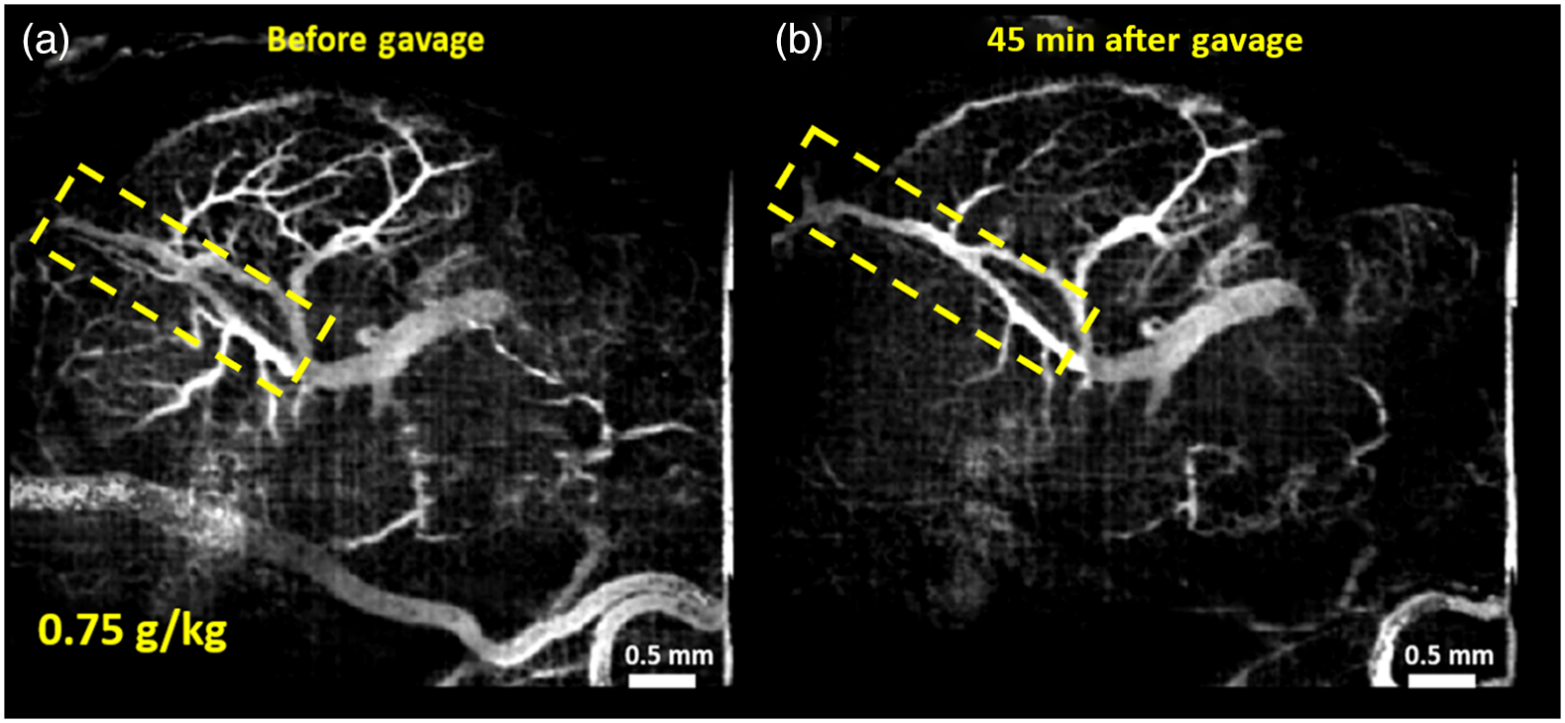
MIP of 3D cm-OCA images of fetal brain vasculature (a) before and (b) 45 min after maternal exposure to ethanol at a dose of 0.75  g/kg. The dashed yellow rectangle shows the main branch of the vessel on which quantifications were made. Figures adapted with permission.^42^

**Fig. 2:**
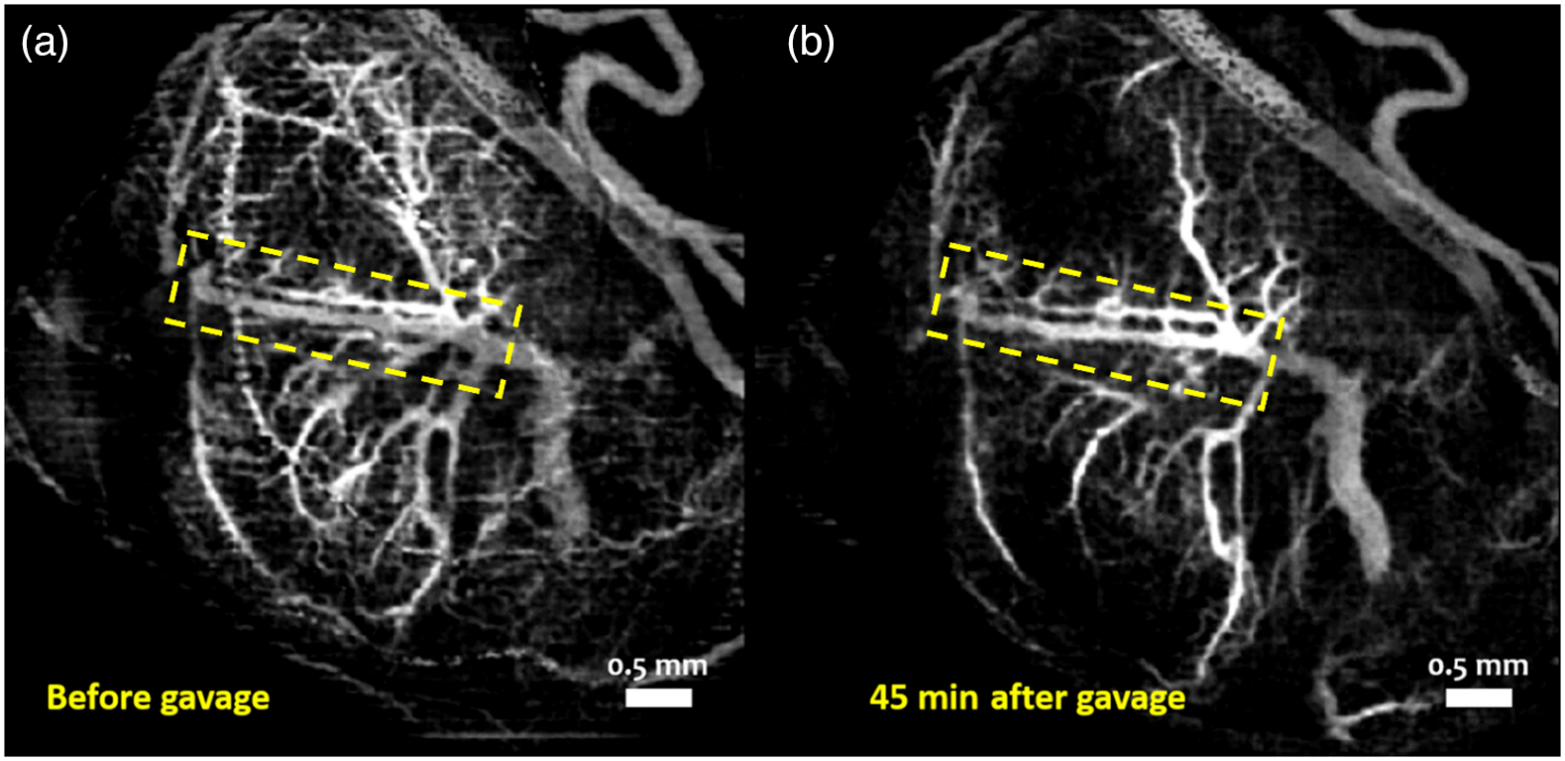
MIP of 3D cm-OCA images of fetal brain vasculature (a) before and (b) 45 min after maternal exposure to nicotine at a dose of 0.1  mg/kg. The dashed yellow rectangle shows the main branch of the vessel on which quantifications were made. Figures adapted with permission.^41^

Vasculature maps shown here are from one representative sample from each of the groups. Figures 1(a) and 1(b) show the MIPs of 3D cm-OCA images before and 45 min after maternal exposure to ethanol at a dose of 0.75  g/kg. Figures 2(a) and 2(b) show the MIPs of 3D cm-OCA images before and 45 min after maternal exposure to nicotine at a dose of 0.1  mg/kg. A slight vasoconstriction can be seen in both these cases at 45 min after maternal exposure to the respective drug.

**Fig. 3:**
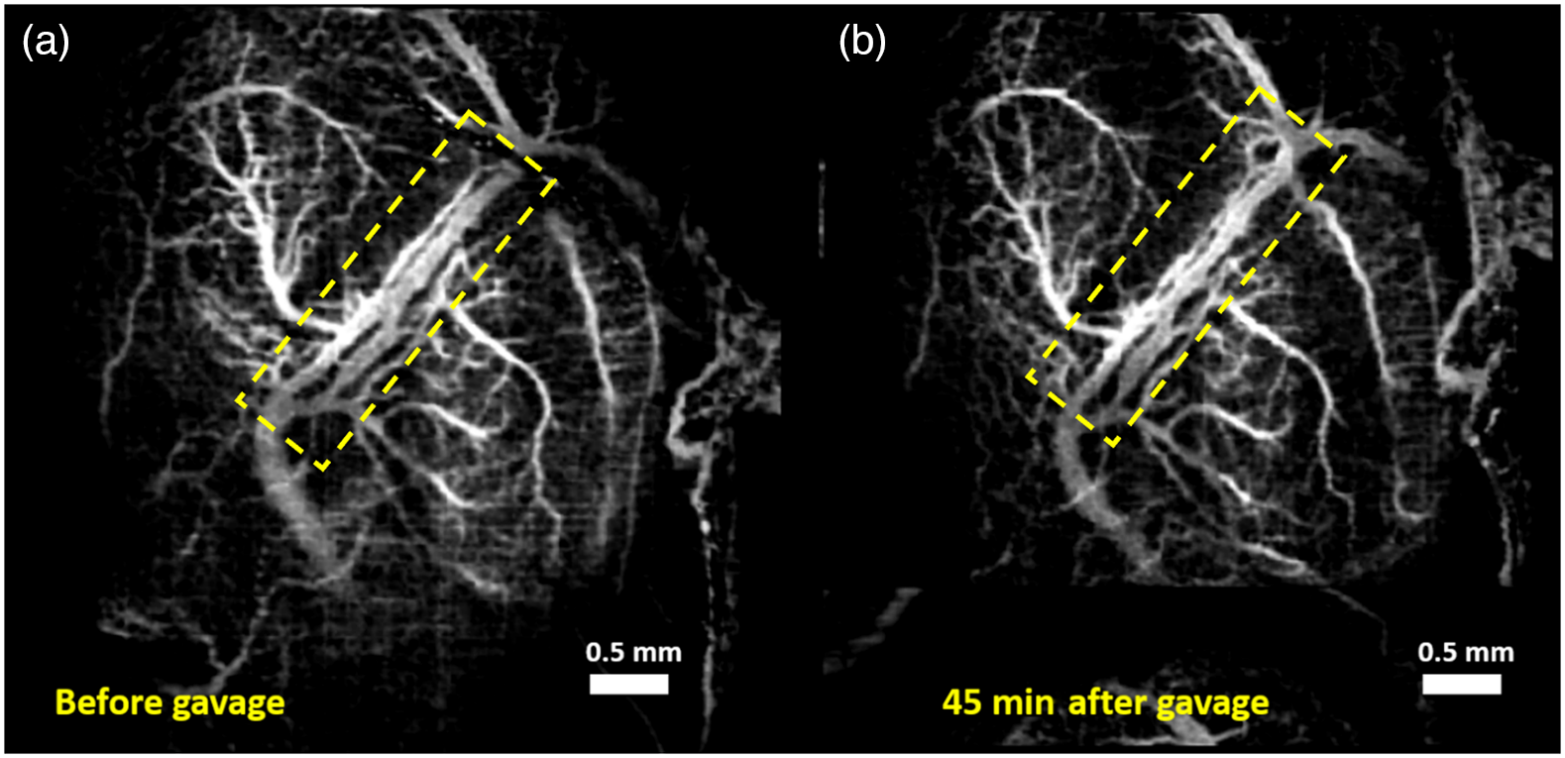
MIP of 3D cm-OCA images of fetal brain vasculature (a) before and (b) 45 min after maternal exposure to a combination of ethanol and nicotine at a dose of 0.75  g/kg and 0.1  mg/kg, respectively. The dashed yellow rectangle shows the main branch of the vessel on which quantifications were made.

Figures 3(a) and 3(b) show the MIPs of 3D cm-OCA images before and 45 min after exposure to a combination of ethanol and nicotine, respectively. Compared to results from exposures to the individual drugs, there is no visible change in the vasculature 45 min after exposure.

**Fig. 4:**
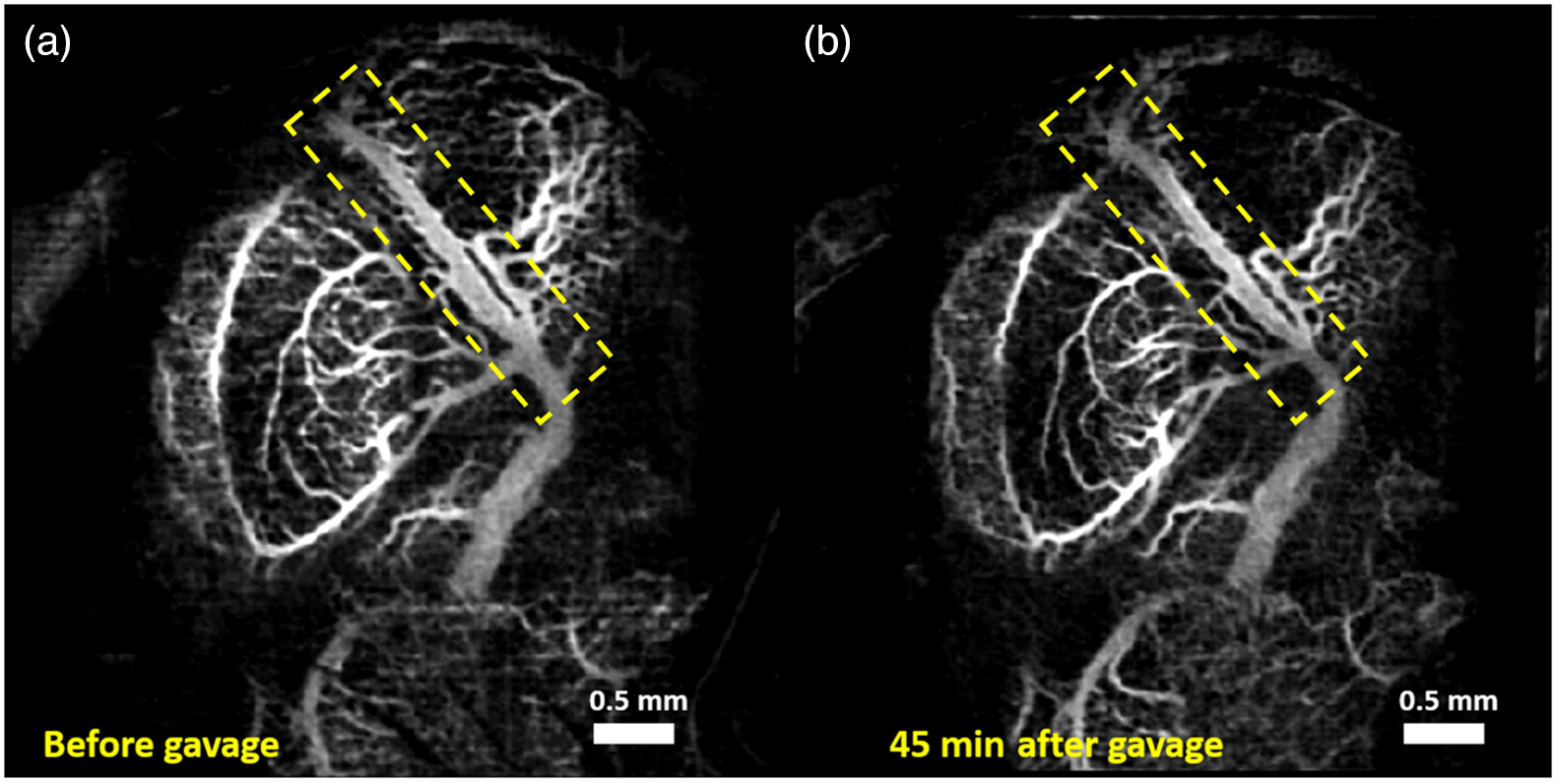
MIP of 3D cm-OCA images of fetal brain vasculature (a) before and (b) 45 min after maternal exposure to a combination of ethanol and varenicline at a dose of 0.75  g/kg and 0.1  mg/kg, respectively. The dashed yellow rectangle shows the main branch of the vessel on which quantifications were made.

Figures 4(a) and 4(b) show the MIPs of 3D cm-OCA images before and 45 min after exposure to a combination of ethanol and varenicline (replacing nicotine), respectively. Similar to results from the ethanol and nicotine group, no drastic change in vasculature was seen 45 min after maternal exposure to ethanol and varenicline.

**Fig. 5:**
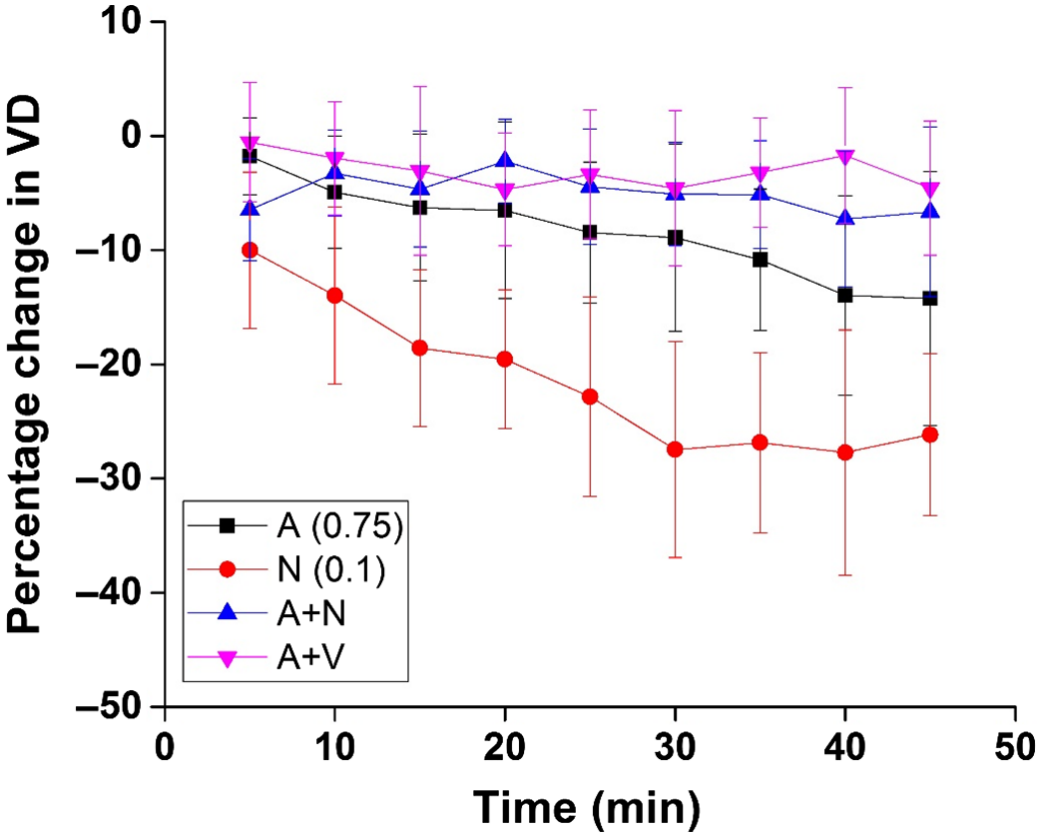
Percentage change in VD after maternal exposure to the respective drugs every 5 min for 45 min. A, alcohol (ethanol); N, nicotine.

Figure 5 depicts the percentage change in VD over a period of 45 min at 5-min intervals. Every sample from every group was used for these calculations. The data represented here are the inter-sample mean and standard deviation. These results show a slight vasoconstriction in the ethanol and nicotine individual groups, whereas there is almost no change in vasculature in both combination groups. The results of the Kruskal–Wallis ANOVA are summarized in Table 1. The P values in bold indicate statistical significance (P<0.05).

**Table 1:** Results of the Kruskal–Wallis ANOVAs. P values in bold indicate statistical significance (P<0.055).

	Degrees of freedom	$\chi^{2}$	P
A	8	31.58	$1\times 10^{-4}$
N	8	61.14	$2.8\times 10^{-10}$
A + N	8	10.63	0.22
A + V	8	10.15	0.25

**Fig. 6:**
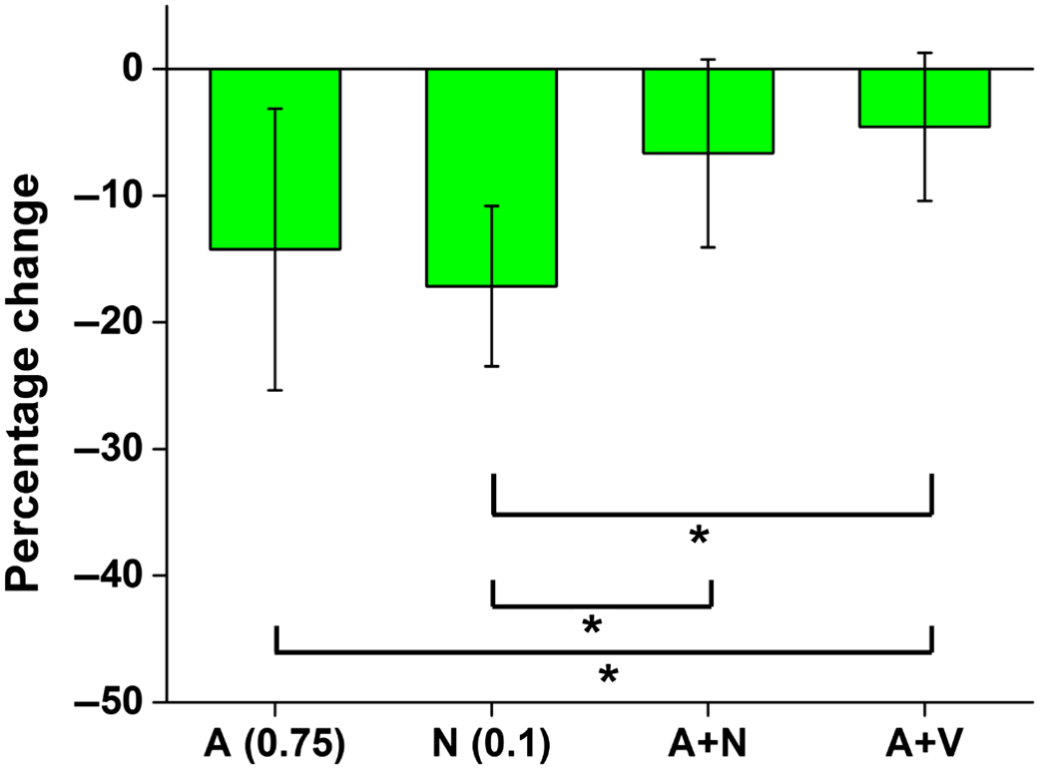
Comparisons of the percentage change in VD at 45 min after maternal exposure to the respective drugs. The asterisk indicates statistical significance by a two-sided Mann–Whitney U test. A, alcohol (ethanol); N, nicotine.

Figure 6 depicts the comparison of percentage change in VD at 45 min after maternal exposure in all 4 groups. A two-sided Mann–Whitney U test was performed between each pair of groups to assess statistical significance. A statistically significant difference (P<0.0083 after Bonferroni correction) was seen between the ethanol group and the ethanol and nicotine combination group, the nicotine group and ethanol and nicotine combination group, and the nicotine group and ethanol and varenicline combination group.

Table 2 summarizes the results of the Mann–Whitney U test. P values in bold indicate statistical significance.

**Table 2:** Results of the Mann–Whitney *U* test. P values in bold indicate statistical significance.

Test	n1	n2	U	P
A versus N	15	18	158	0.13822
A versus A + N	15	15	74	0.11483
A versus A + V	15	20	64.5	**0.00458**
N versus A + N	18	15	34	$7.26\times 10^{-4}$
N versus A + V	18	20	20	$8.93\times 10^{-6}$
A + N versus A + V	15	20	127	0.45313

## 4 Discussion

Most non-medical drug users have the tendency to abuse multiple substances at once or consecutively.^46^^–^^48^ Poly-drug use is of serious concern because it is associated with a unique set of side effects and complications,^49^ which could be caused by various biochemical processes occurring in the body simultaneously after consumption, including synergy,^50^ cross-tolerance, and additive effects.^51^ Compared to single-drug use, poly-drug use has resulted in a greater number of traffic accidents,^52^ greater levels of psychomotor impairment,^53^ higher toxicity,^54^ and a higher likelihood of death due to overdose.^55^^,^^56^ However, its effect on fetal development is far less studied.

The second trimester of human gestation is a crucial period for fetal neurogenesis and angiogenesis. Our previous work has shown that maternal exposure to teratogens during this period causes drastic changes in fetal brain vasculature.^39^^–^^42^ However, this study, for the first time, reports the changes in developing brain vasculature after maternal exposure to a combination of ethanol and nicotine simultaneously. We utilized cm-OCA to obtain vasculature maps of the fetal brain before and after exposure to both ethanol and nicotine. Results were quantified and compared to results from previous studies where the maternal exposure was only to ethanol or nicotine independently. Results showed that there was no significant change in vasculature in the group with combined exposure compared to the individual drug groups where their vasoconstriction was observed.

Smoking cessation is difficult due to the highly addictive behavior of nicotine. Nicotine imitates the function of the neurotransmitter acetylcholine by binding with the nicotinic acetylcholine receptors in the brain. This causes a release of dopamine in the brain, which in turn leads to a reduction in nicotine withdrawal symptoms.^57^ This mechanism is exploited in nicotine replacement therapy, where nicotine in low doses is delivered over a period of a few minutes compared to the higher doses obtained in a few seconds through smoking.^58^ Varenicline, on the other hand, is a partial agonist and stimulates receptors at a lower level than nicotine. It is highly selective and binds only to the α4β2 receptors rather than other common nicotinic receptors. Varenicline decreases cravings and withdrawal symptoms and lowers the stimulation of the mesolimbic dopamine system that is associated with nicotine addiction. It can significantly prevent both short-term and long-term relapse.^43^ Due to this, varenicline has quickly become the first drug of choice for smoking cessation. Hence, in this study, we also chose to replace nicotine with varenicline at a similar dose and test the effects of its combined exposure with ethanol on fetal brain vasculature. Results showed no drastic change in vasculature after exposure to ethanol and varenicline, similar to results from the group that was exposed to a combination of ethanol and nicotine. This study showed that ethanol and nicotine exert antagonistic effects on developing fetal brain vasculature. Balaraman et al.,^59^ showed similar results where ethanol and nicotine exerted mutually antagonistic effects on fetal neuronal stem cell development. They also showed that nicotine, at concentrations attainable in the circulation of cigarette smokers (dose used in this study), induced a more than four-fold increase in all of the ethanol-suppressed microRNAs (miRNAs). However, at higher doses, a dose-related decline in miRNA expression was observed. Since we noticed similar effects to Balaraman et al. at human doses, our future work will involve testing pharmacologic doses to evaluate if changes in vasculature follow similar patterns to miRNA expressions. Future work will focus on assessing whether this change is transient or more permanent.

The doses selected for this study were 0.75  g/kg of ethanol and 0.1  mg/kg of nicotine and varenicline. These doses were chosen based on our previous dose-response studies,^41^^,^^42^ where these two doses showed moderate changes in vasculature compared to higher doses. All VD quantifications were made on the main branch of the vessel, indicated by the yellow dashed rectangle. This was done to reduce the influence of external factors, such as maternal heartbeat and respiration and the effects of clamping the uterus and anesthesia.

As mentioned in our previous publications,^40^^–^^42^ limitations to our current technique include system sensitivity and sensitivity roll-off that affect the phase stability. This could, in turn, affect the quality of the cm-OCA vasculature map, particularly for deeper vessels. Apart from orienting the fetus such that the dorsal vessels were clearly visible to improve sensitivity in this study, our future work will involve a projection-resolved algorithm^60^ to reduce shadowing artifacts and image deeper vessels, a phase correction scheme,^37^^,^^61^ a 2D Gabor wavelet filter,^62^ and faster imaging speeds to reduce artifacts due to bulk motion. We are also implementing fluorescent microscopy techniques to corroborate the cm-OCA results.

## 5 Conclusion

This study assessed the effects of combined maternal exposure to ethanol and nicotine on fetal brain vasculature using cm-OCA *in utero*. Results from combined exposure groups were compared to groups with single-drug exposure from previous studies. While vasoconstriction was noticed in groups with independent ethanol and nicotine exposure, the groups with combined exposure showed no drastic change in vasculature. Nicotine was replaced with varenicline in one of the combined groups. Results were similar to the group exposed to a combination of ethanol and nicotine.
